# Supplementary material for: Are carbon emissions trading and green financial instruments synergistic? -Comprehensive quantitative research based on content analysis
Source: PLoS One. 2024 Mar 7;19(3):e0298601. doi: 10.1371/journal.pone.0298601 (PMC10919601; doi:10.1371/journal.pone.0298601)
Supplement: S1 Data — (ZIP) [file pone.0298601.s001.zip › Data/地级市Z_F工作报告.zip!/║■▒▒/╗╞╕╘╩╨/└┤╫╘╣1⁄2╓┌║┼í╛┬φ┐╦╩2╛▌═°í┐_2017.docx]

各位代表：

　　现在，我代表市人民政府向大会报告政府工作，请予审议，并请市政协各位委员和其他列席人员提出意见。

　　一、过去五年的工作回顾

　　本届政府任期五年来，在市委的坚强领导下，在市人大、市政协的监督支持下，高举“四个大别山”发展旗帜，大力实施开创战略，突出“双强双兴”发展重点，攻坚克难，加快发展，较好完成了市四届人大一次会议确定的各项任务，实现了“三年明显变化、五年大变化”发展目标。

　　经济总量稳步提升。预计2016年实现地区生产总值1720亿元，年均增长9.4%;固定资产投资2050亿元，年均增长21%;规模以上工业增加值年均增长11.5%;一般公共预算收入119.5亿元，年均增长17.4%;社会消费品零售总额达到969亿元，年均增长12.8%;外贸出口总额64500万美元，年均增长20.3%;金融机构存贷款余额分别达到2700亿元和1171亿元，年均增长18%、20%。固定资产投资、一般公共预算收入、外贸出口总额、金融机构存款余额等指标实现翻番。县域经济竞相发展，麻城、武穴、浠水、蕲春、黄州5个县市区生产总值过200亿元，武穴、麻城、红安、蕲春、黄梅5个县市一般公共预算收入过10亿元。

　　产业结构持续优化。三次产业比由2011年的27.75：38.92：33.33调整为22.75：38.67：38.58。农业综合生产能力稳定增强，农业结构不断优化，新型经营主体快速壮大。建成千亩以上现代农业示范园128个，武穴、黄梅、团风农产品加工园列入省农业产业化示范园区。规模以上农产品加工企业达到521家，全口径农产品加工产值与农业总产值比值为1.65:1。新型工业化步伐加快，规模以上工业企业达到1497家。国家级黄冈高新区创建进入审批阶段。高新技术企业达到177家，高新技术产业增加值占GDP比重达到8.9%，获评全国科技进步城市。服务业活力增强，文化产业、金融业长足发展，旅游业步入全省第一方阵。旅游人数和综合收入连年保持20%以上增长，红安、罗田、麻城、英山等县市获评湖北旅游强县，并入选国家全域旅游示范区创建名单，全市4A景区达到15家，成功创建首个中国漂流谷，龟峰山风景区通过5A景观质量评审。大别山世界地质公园创建工作顺利推进。红安、麻城、英山、黄梅、罗田、蕲春等县市列入全国电子商务进农村综合示范县。

　　基础设施不断完善。交通建设投入465亿元，黄冈长江大桥、武冈城际铁路、黄鄂高速、麻阳高速、大别山红色旅游公路及支线、黄冈大道等重大交通项目建成通车，高速公路里程突破700公里，公路总里程达到2.87万公里，实现县县通高速、乡乡通省道、村村通客车。江北铁路、武穴长江大桥等项目进展顺利。水利建设投入162.1亿元，完成39条中小河流综合治理、782座水库和57公里回水堤防整险加固。电网建设投资69.8亿元，农村电网加快改造升级。开展国家智慧城市、数字城市试点，基础通讯升级换代，城区百兆光纤网络覆盖率100%。

　　城乡面貌明显改观。完善“四线”控制规划，城乡建设投资额是前五年的4倍，城镇化率提高7.61个百分点、达到44.52%。市区面积由28平方公里扩大到50平方公里，完成101公里主次干道黑化亮化。遗爱湖公园成功创建国家湿地公园，获评湖北十大最美湖泊，极大提升了城市品位。总规划面积63平方公里的白潭湖片区初具雏形。各县市“双十支撑、两区互动”加快推进，卫生城市、文明城市等创建工作掀起热潮，红安“一河两岸”、麻城孝感乡文化公园、黄梅滨河新区等成为城市风景。黄团浠区域协同发展启动实施，武穴城乡一体试点扎实推进，24个国家重点镇、21个省级文化小镇、13个省级重点中心镇、10个省级特色镇、111个省级美丽宜居村庄、200个“一带一片”美丽乡村试点、26个中国传统村落建设梯次推进，城乡面貌发生深刻变化。实施“天蓝、地绿、水碧、土净”工程，强力推进“雷霆行动”，在全省率先完成“大气十条”任务，连续多年超额完成节能减排目标，省级生态市创建取得实效。

　　改革开放深入推进。大别山革命老区振兴发展上升为国家战略。“363”“2022”重点改革项目有序推进，“放管服”改革成效明显，取消、调整和下放市级行政审批事项254项，建立投资项目联审联批平台，推行基本建设项目收费“一表制”。颁发全省首张农村土地承包经营权证，全面实施“五证合一”“一照一码”。拓宽投融资渠道，新设立50亿元黄冈大别山绿色发展股权投资基金、12亿元中小企业转贷基金，新引进股份制商业银行6家，336家企业在各类资本市场挂牌上市。国有农场、国有林场及农村各项改革全面推进，户籍制度改革、营改增等财税体制改革、公车改革、龙感湖农场改革、白莲河水库管理体制改革稳步实施，公安改革、供销社综合改革、用地增减挂钩试点、领导干部自然资源资产负债表编制和审计工作试点进展顺利。浠水国家级转型升级示范开发区建设、小池省级全面深化改革示范区建设、“临江四城”开放开发成效彰显。以市校合作为载体的人才科技创新、以大别山金融工程为载体的金融创新、以市场主体增量提质工程为载体的创业创新取得突破。对接大武汉，园区共建、承接产业转移卓有成效。与黄石、九江跨江合作，与信阳、安庆、六安等环大别山一体化合作有力推进。招商引资累计引进项目2417个，完成投资2775亿元。中国光彩事业黄冈老区行签约项目245个，开工191个，已投产76个。国内最大的LNG工厂昆仑能源建成投产，黄冈历史上最大的工业单体项目晨鸣林浆纤一体化开工建设。

　　社会事业全面进步。公共财政支出八成以上投向民生领域，养老保险、医疗保险等社会保障基本实现全覆盖。累计有55万困难群众享受城乡低保，5.76万农村五保对象得到供养，11万残疾人领取生活补助，1.99万户住房困难家庭入住保障性住房，220万农村居民饮水安全问题得到解决，21.66万被征地农民参加养老保险，完成农村危房改造8.88万户，实施各类棚户区改造11.5万户，城镇新增就业36.4万人、20.1万人实现再就业。深入推进精准扶贫“1351”行动计划，贫困地区生产生活条件不断改善，累计减少贫困人口40万人。城镇和农村常住居民人均可支配收入达到24650元、11150元，分别是2011年的1.7倍、2.1倍。建成黄冈老年大学、博物馆、黄梅戏大剧院、职教园、体育中心等民生工程。中小学校办学条件明显改善，所有县市区通过义务教育发展基本均衡国家评估验收，教育质量实现恢复性增长。深化医改和计划生育服务管理改革，卫生资源总量翻番，互联网+计生+妇幼健康服务模式全面推行。文化事业蓬勃发展，群众文化生活丰富多彩，电影《青春派》获全国“五个一”工程奖，多部精品戏曲进京演出。文明创建深入实施，获评省级文明城市。成功创建中华诗词之市、中国书法城、中国东坡美食文化之乡，成功举办全国文化科技卫生“三下乡”集中服务活动、市四运会和“挺进大别山”系列体育赛事。创新阳光信访、法务前沿、“1343”维稳机制、网格化管理和立体化防控模式，法治黄冈、平安黄冈建设成效明显。深入实施“六大食品安全示范工程”，食品药品监管不断加强。安全生产连年获全省考核优秀等次。和谐企业创建取得积极成果。

　　自身建设日益加强。扎实开展党的群众路线教育实践活动、“三严三实”专题教育和“两学一做”学习教育，与时俱进加强政府自身建设。自觉接受人大、政协监督，办理人大代表建议611件、政协提案1587件。完成“六五”普法，启动“七五”普法。推进法治政府建设，实现“三个清单”制度、政府决策法治化、以城市管理为主的行政执法体制改革、行政复议体制机制改革和政府法律顾问制度全覆盖。严守党的政治纪律和政治规矩，贯彻执行中央八项规定，认真落实全面从严治党主体责任，旗帜鲜明反腐倡廉，严肃查处各类违纪违法案件，创新“廉洁预案、廉洁备案”管理，加强权力运行监督制约，强化效能监察和审计监督，政府系统廉政建设不断加强。建设工程、公共资源交易、政府采购等重点领域预防腐败机制进一步完善。切实抓好巡视反馈问题和百姓问政、行风热线曝光问题的整改，深入推进行风评议，政风行风持续好转。

　　国防动员、国家安全、人民防空、民兵预备役、优抚安置等工作进一步加强。与工会、共青团、妇联、工商联、科协等群团组织联系进一步密切。对台、对口援疆、民族宗教、外事侨务、残疾人事业、妇女儿童事业、移民、农垦、老龄、统计、保密、档案、气象、地震、方志、公积金管理、机关事务管理等工作都取得新的成绩。

　　刚刚过去的2016年，我们积极应对特大洪涝灾害和经济下行压力，全力“一抗四保”，合力“三大决战”，保持了稳中有进、进中向好的发展态势。预计地区生产总值同比增长7.6%，固定资产投资增长14%，规模以上工业增加值增长8.5%，社会消费品零售总额增长10%，一般公共预算收入增长5.7%(同口径增长11%)，城镇和农村常住居民人均可支配收入分别增长9.0%、8.8%。扎实推进“四大行动”。120个“双百”项目开工，完成投资360亿元。成功争取京九客专阜九段、黄冈至黄梅铁路、合安九高铁黄梅段等重大项目进入部省规划。新引进307个招商重点项目，完成投资560.6亿元。“千企联百校”签约项目140个。五类市场主体总量达到44.9万户，居全省第二。扎实推进供给侧结构性改革。“三去一降一补”取得实效，完成钢材、水泥等59种过剩产品产能压减任务，清退“四上”企业39家，可售商品住房去化周期在可控范围内。全市共减免税收15.7亿元。重点领域改革稳步实施。扎实推进脱贫攻坚。预脱贫453个村、30.04万人，易地扶贫搬迁10886户、20173人，“五位一体”产业扶贫模式在全国推广，全国“万企帮万村”精准扶贫行动现场会在我市召开。扎实推进政府效能建设。践行实干作风，强化责任担当，政府决策提速，行政效率提高，执行力和公信力进一步提升。成功举办首届黄冈楚商大会、大学生创业节、大别山金融论坛、国家千人计划专家黄冈行、东坡文化节暨黄梅戏艺术节等重大活动。成功创建国家园林城市，国家卫生城市、省级环保模范城市创建通过初步验收。军政军民融合发展，第三次获评全国双拥模范城。

　　过去一年的历程极不平凡，收获极为珍贵。全市人民风雨同舟、严防死守、以钢铁般的意志战胜超历史的洪水，迎难而上、自强不息、以大无畏的气概战胜创历史的灾害，夺取了抗洪救灾的重大胜利。“万众一心、众志成城、不怕困难、顽强拼搏、坚韧不拔、敢于胜利”的抗灾精神，必将激励我们在老区振兴发展的新征程中砥砺奋进，阔步前行!

　　五年的实践告诉我们，做好政府工作，必须坚持五大发展理念、紧扣第一要务不动摇，必须坚持深化改革、扩大开放不动摇，必须坚持依法行政、执政为民不动摇，必须坚持从严治党、实干兴市不动摇。

　　五年的成绩来之不易，得益于省委、省政府和市委的正确领导，得益于全市人民团结拼搏、共同努力，得益于历届市委、市政府打下的良好基础，得益于市人大、市政协的有力监督和支持。在此，我代表市人民政府，向全市人民，人大代表、政协委员，向各民主党派、工商联、无党派人士、人民团体、离退休老同志，向驻黄部队、武警官兵、政法干警、部省在黄单位，向所有关心支持黄冈发展的同志们、朋友们，表示崇高敬意和衷心感谢!

　　在看到成绩的同时，我们也清醒地认识到：发展不够依然是黄冈最大的实际，经济总量不大、人均水平较低、对外开放不够、市区首位度不高等问题比较突出;产业发展短板明显，结构不优、质效不高、工业腿短，转型升级任重道远;如期脱贫、同步小康任务艰巨;区域发展不平衡，财政收支矛盾突出;少数干部不会为、不作为、慢作为现象依然存在，政府效能有待进一步提高。对此，我们要保持清醒头脑，采取有效措施，切实加以解决。

　　二、今后五年的奋斗目标

　　加快黄冈振兴崛起，其时已至，其势已成!今后五年，多项重大国家战略在黄冈实施，多重政策利好在黄冈叠加，多种要素资源在黄冈聚集，新一轮开放开发的浪潮激活黄冈，新一轮区域经济格局的调整提升黄冈，我们将迎来一个充满期待、大有作为的战略机遇期。全民向往的小康梦、振兴崛起的老区梦，汇聚成黄冈发展的强大气场，凝结成振兴发展的巨大力量。只要我们顺势而为，乘势而上，就一定能够赢得主动、赢得优势、赢得未来。

　　今后五年政府工作的指导思想是：高举中国特色社会主义伟大旗帜，全面贯彻党的十八大和十八届三中、四中、五中、六中全会精神，以马克思列宁主义、毛泽东思想、邓小平理论、“三个代表”重要思想、科学发展观为指导，深入学习贯彻习近平总书记系列重要讲话精神，统筹推进“五位一体”总体布局和协调推进“四个全面”战略布局，认真践行五大发展理念，贯彻落实市第五次党代会决策部署，加快振兴崛起、决胜全面小康，为实现第一个百年奋斗目标谱写黄冈发展新篇章。

　　今后五年的主要任务是：

　　——把握战略机遇，实现综合实力新跨越。坚持稳中求进，好中求快，奋勇争先，继续实施“双强双兴”，扎实推进“四大行动”，经济增速高于全国全省平均水平，全面完成“十三五”规划，实现总量增加、质效提升、动能转换、结构优化、特色鲜明，成为全省“一元多层次”战略体系的重要增长极，引领大别山革命老区振兴发展。努力把黄冈建成欠发达地区科学发展示范区、全国重要的粮食和特色农产品生产加工基地、长江中下游地区重要的生态屏障、全国重要的旅游目的地。

　　——敢于先行先试，增创改革开放新优势。秉承敢为人先的老区传统，用足先行先试的政策优势，解放思想，大胆实践，坚持问题导向，务实推进改革，增创体制机制新优势。推进更深层次更高水平双向开放，积极融入武汉城市圈和长江中游城市群，对接长江经济带和湖北自贸区，推动环大别山市际战略合作，大力发展外向型经济。争创国家创新型城市，打造大别山金融工程升级版、市校合作升级版和区域协作升级版。

　　——注重转型升级，推动产业发展新突破。加快构建以新型工业为主导、现代农业为基础、现代服务业为纽带的产业体系，打造振兴崛起的强劲引擎。贯彻“中国制造2025”，实施工业倍增计划，推动黄冈工业转型升级。坚持“两转四用”，优化农业产业体系、生产体系、经营体系，提高土地产出率、资源利用率、劳动生产率，促进农业提质增效，实现传统农业大市向现代农业强市迈进。实施服务业提速升级行动计划，把文化旅游业作为战略性支柱产业，建成主客共享、快达慢游的旅游目的地，实现旅游综合收入年均增长20%。提升创新能力，促进科技成果转化和产业化，高新技术产业增加值占GDP比重达到16%以上。实施“互联网+”行动计划，促进新一代信息技术与制造业深度融合，推动产业跨界融合，让新技术、新产品、新业态、新模式迸发涌流。

　　——加强城乡统筹，建设宜居宜业新黄冈。顺应发展规律，统筹“三大结构、三大环节、三大动力、三大布局、三大主体”，构建和谐宜居、富有活力的新型城镇化体系。努力建设省际区域性中心城市，形成以市区为核心、县市城区为支撑、示范镇为节点、新农村和新型社区为依托的“四位一体”新型城镇化格局，城镇化率达到52%左右。升级“一区两带”战略布局，促进区域联动发展、协调发展。

　　——坚持绿色发展，树立生态文明新样板。坚持生态立市、绿色崛起，推进“五化”协同发展。执行主体功能区规划，构建“六区三园”红线保护、“一江六水”生态廊道为架构的生态格局，加快形成绿色发展方式和生活方式，努力打造全国生态文明建设示范区，创建国家级生态市，让黄冈天更蓝、地更净、水更清、山更绿、空气更清新。

　　——决胜全面小康，开创民生幸福新时代。坚决打赢脱贫攻坚战，全市建档立卡贫困人口全部脱贫、贫困村全部出列、贫困县全部摘帽，达到“两不愁、三保障”综合标准。城乡居民收入稳步提升，覆盖城乡的社会保障和公共服务体系更加完善，文化软实力不断增强，社会文明显著进步，全面建成法治政府。把人民健康放在优先发展的战略地位，把健康政策融入全局，打造健康中国的黄冈样本。对照全面小康5大方面39项指标，补齐短板，确保老区人民与全国人民一道进入全面小康社会。

　　三、2017年工作安排

　　2017年全市经济社会发展主要预期目标为：地区生产总值增长8%左右，规模以上工业增加值增长8.5%，固定资产投资增长14%，社会消费品零售总额增长11%，外贸出口总额增长10%，一般公共预算收入增长8%，居民收入增长与经济增长基本同步，居民消费价格指数涨幅控制在3.5%以内，城镇登记失业率控制在4.5%以内，完成省定节能减排目标。在实际工作中，努力争取更好结果。

　　围绕上述目标，重点抓好以下七个方面的工作：

　　(一)以稳增长为首要任务，保持经济健康发展。数量经济是效益经济的基础。只有做实增量，才能做大总量;只有保持快的速度，才能巩固稳的态势。要坚持稳中求进总基调，促进经济平稳较快发展。

　　发挥投资关键作用。把扩大有效投资作为稳增长的主动力，加快制订有效投资三年滚动计划。全年完成固定资产投资2300亿元以上。强化财政资金撬动和金融信贷保障，充实产业引导资金，助推经济发展。落实创业投资政策激励，激活民间投资，力争民间投资比上年净增150亿元以上。建立PPP“三库一平台”，推进PPP建设取得突破。培育新的消费热点，深入实施“十大扩消费行动”，实现消费对经济增长的贡献率稳步提高。鼓励企业“走出去”，巩固船舶、生物医药等省级出口基地，加快板栗、蕲艾等10家传统农产品出口企业转型升级，支持祥云集团、宏源药业等10家骨干型出口企业做大做强。

　　发挥项目支撑作用。开展项目攻坚年活动。强力推进“双百”项目，加快晨鸣林浆纤一体化、大别山电厂二期、祥云合成氨、伊利酸奶、恒天文化产业城、光谷联合科技城、际华纺织、龙源石膏、襄大农牧等重点项目建设。加大江河湖水系连通、白莲河水库水生态治理、鄂黄第二过江通道、长江崩岸治理、主要支流及中小河流治理、华阳河蓄滞洪区建设、城市防洪等项目谋划和推进力度。推进黄冈至黄梅铁路、合安九高铁黄梅段、江北铁路、武穴长江大桥、麻竹延长线、长江深水航道整治工程蕲春段、陶店至巴河一级公路、团黄大道扩建等项目建设。全力抓好灾后重建基础设施项目对接和落地，用三年时间将易涝县市排涝能力提高一倍。推进数字城市和智慧城市试点，实施宽带乡村惠民工程。加快新一轮农村电网改造升级，全面消除存量“低电压”问题。市区重点抓好稳健医疗等十大改扩建项目，欧派定制家具等十大招商项目，湖北绿宇环保等十大新建项目，中船重工军民融合产业园等十大续建项目，实现当年完成投资300亿元以上。

　　发挥园区聚集作用。以建设国家级高新区为契机，推动高新区体量扩张、功能扩充、实力扩展、贡献扩大。进一步优化市区及各县市园区的产业布局和功能定位，形成优势互补、各具特色的差异化发展格局。提升园区承载能力，增强投资吸引力，推进“满园行动”。进一步规范项目入园、建设、投产、运营及退出程序，提高投资强度、单位产出率和贡献率。加快推进武汉经开区黄冈产业园、光谷黄冈科技园、黄州火车站经济开发区、南湖产业园建设。支持团风发展临港经济示范区，支持红安创建国家产城融合示范区，支持麻城、黄梅创建省级高新区，支持浠水国家级转型升级示范开发区建设，支持蕲春打造大健康产业千亿园区，支持武穴建设新能源汽车制造基地，支持龙感湖打造中部地区纺织面料生产基地。

　　发挥实体经济基础作用。聚焦实体经济，引导各类要素“脱虚入实”。深化服务企业行动，深入开展“六抓六促”活动，切实解决实体经济特别是中小微企业发展难题。推进环境再造，强化环境就是生产力、安商重于招商的理念，多服务、不干扰，多关心、不指责，建设法治化的市场营商环境，打造服务高地、要素洼地。营造尊重创新创造、尊崇企业家的浓厚氛围，以企业稳带动实体稳、企业家强带动经济强。

　　(二)以转型升级为主线，提升发展水平。没有强大的产业，就没有黄冈的振兴崛起。新常态下，必须一手抓传统产业改造升级，一手抓战略性新兴产业培育，打造经济增长的双引擎。

　　加快工业转型发展。强化工业是立市之本、强市之基的共识，突出工业主导地位。实施工业倍增计划。坚持巩固存量与扩大增量并重，顶天立地与铺天盖地并重。当年完成工业投资占总投资的40%以上，新进规企业突破100家。促进工业创新发展。围绕转型升级、提质增效目标，实施工业+高校科研院所、+信息化、+品牌质量行动。推动企业与高校科研院所深度合作，加大企业研发投入，启动新一轮技术改造工程，加快传统产业智能化改造，着力开发新技术、新工艺、新产品。加快行业信息化服务平台建设，推进信息技术在企业管理、产品研发、市场营销环节的应用，增强企业核心竞争力。推行质量品牌提升计划，发扬“工匠精神”，加强品牌建设，培育“百年老店”。提升优势产业。围绕千亿产业培育目标，整合资源链、产业链、创新链、人才链，促进食品饮料、纺织服装、医药化工、新型建材、机械电子等优势产业集群发展、稳步迈向中高端。做大做强窑炉、中医药健康产业、汽车配件等重点成长型产业和31个特色产业集群，打造一批百亿产业。壮大骨干企业。实行“一企一策”，跟踪服务。支持昆仑能源、科峰传动、索菲亚、青江化工、兴和股份、鸿路钢构、李时珍集团、迅达药业、山河集团、长安集团等企业发展，鼓励龙头企业强强联合、兼并重组，成为行业领军企业。培育新兴产业。围绕高端装备、新能源等十大重点领域，加快发展战略性新兴产业。规划建设一批新兴产业聚集基地和孵化器，组建一批产业创新联盟，设立产业投资基金，培育新经济，发展新动能。

　　加快现代农业提质发展。坚持用新的理念引领现代农业发展，推进农业供给侧结构性改革，着力提升产能、调优结构、提高质效、激活市场。发展规模经营。细化和落实承包土地“三权分置”办法，培育新型农业经营主体和服务主体。深化农村产权制度改革，搭建市县乡三级农村综合产权交易平台，引导发展适度规模经营。不断壮大村级集体经济，千方百计增加农民收入。发展生态农业。推进标准化、绿色化、品牌化生产，发展设施农业、减灾农业。严守耕地红线，推进高标准农田建设，藏粮于地、藏粮于技，保护和提高粮食综合生产能力。实施科技兴农战略，提高农业良种化、机械化、科技化水平，保证农产品安全，争创农业标准化示范区。着力构建粮经饲统筹、农林牧渔结合、种养加一体的产业体系。加快地标品牌产业化步伐，扩大粮油、蔬菜、水产、茶叶、药材、畜禽等优质农产品供给。推进农业“接二连三”，打造一批健康养生农业、休闲观光农业样板，创新发展定制农业、创意农业。发展农产品加工业。大力发展农产品精深加工和副产品综合利用，延长农业产业链价值链。推进地标产品集约化、规模化、基地化生产，支持发展地标产品加工园区。

　　加快现代服务业提速发展。提升生活性服务业，注重引进城市商业综合体，培育特色商业街区，改造建设大型专业市场，完善农村和社区商业网点，加快构建现代城乡商贸服务体系。支持黄商集团实施“线上线下千店计划”。壮大生产性服务业，以物流基础设施和物流公共信息平台为支撑，推进专业物流园区发展。加快港口物流园建设，大力发展水运物流。支持黄州区打造全功能港区。培育消费性服务业，促进旅游与文、商、体、农、康融合发展，突出全域旅游，发展智慧旅游，整合全市资源，加快建设景区之间通道，构建信息互通、线路互联、优势互补、客源互送的旅游格局，拥抱全民旅游时代。积极创建国家中医药健康旅游示范区。支持黄梅禅文化旅游区建设，支持红安、罗田、麻城、英山等地加快全域旅游示范区建设，构建新型旅游发展格局。推进现有景区升级，实现5A景区零的突破。引导新兴服务业，大力发展电子商务、信息咨询等基础服务业，规范发展法律服务、会计事务等中介服务业，创新发展文化体育、康养家政等公共服务业。

　　(三)以城乡统筹为路径，建设美好家园。围绕山水、人文、生态，在统筹上下功夫，重点上求突破，个性上做文章，建好城市会客厅，美化农村后花园，推动城乡比翼齐飞。

　　提升市区首位度。完善市区一体开发体制机制，启动新一轮城市总体规划修编，加快黄团浠区域协同发展，更加注重基础先行、人文纽带、产城融合，促进市区扩容提质，着力打造黄冈都市区。实施城市有机更新，推进防洪排涝、棚户区改造、污水处理厂、智慧交通、立体停车场、垃圾综合治理等工程建设，启动地下综合管廊、海绵城市、城市云中心、万达商圈、江滩公园、赤壁公园改造等城市整理提升工程，启动公共自行车共享、新能源汽车充电桩等服务项目。推进公交车、出租车改革，争创省级公交示范城市。加快白潭湖片区发展，积极推进大别山区域医疗中心、综合馆、金融服务港等项目建设，同步推进基础设施建设、招商项目落地和体制机制创新。开展闯红灯、车辆违行违停等问题整治，深入推进“四城联创”和城管会战，提升市区品质。

　　增强县城集聚能力。立足各县市资源禀赋和地方特色，积极拓展县城空间，完善城市功能，彰显城市个性，提升城区吸引力。完善激励政策，加快农业转移人口市民化。坚持分类指导，一县一策，支持县域经济特色发展、错位发展。完善科学发展综合考核体系，形成争先进位、竞相发展的浓厚氛围，力争有县市区进入全省分类考核第一方阵。

　　建设美丽乡村。强化乡村规划引领，全面开展农村环境综合整治，规范农村建房，加强农村危房改造、农村生活垃圾处理、城镇污水处理，开展城乡垃圾分类收集试点，推进“四好农村路”建设。支持罗田率先实现25户以上自然垸通硬化路。支持武穴、英山开展整县推进城乡一体化和美丽乡村建设试点。继续推进小池、七里坪、散花、南湖等“四化同步”示范镇建设，加快推进“临江四城”和24个国家重点镇、50个特色小镇、300个美丽乡村和旅游名镇名村建设。在全市开展“厕所革命”，彻底消灭传统旱厕，建设无公害卫生户厕和公厕。在城乡开展“扫帚行动”，让黄冈点上出彩、线上美丽、面上整洁。

　　(四)以改革开放为引领，激发创新活力。知常明变者赢，守正出新者进。必须坚持改革不停顿、开放不止步、创新不言败，增强老区发展动力和活力。

　　深化供给侧结构性改革。综合运用市场、法治手段，落实“三去一降一补”任务。坚决淘汰落后产能。落实支持农民工等新市民购房政策，推行棚改货币化安置，促进房地产去库存。规范房地产开发、销售、中介等行为，确保房地产市场平稳健康发展。引导企业股权融资、市场化债转股，稳妥降低杠杆率。规范政府性债务和投资管理。加快政府投融资平台市场化改造，增强自我经营能力。深化“放管服”改革，简政放权，减少审批环节，促进减税、降费、降低要素成本。深化行政执法体制改革，探索建立跨部门综合执法、跨区域联动联调机制。深化公安改革、农村综合改革、商事制度改革，持续推进环保体制改革、国有企业改革、供销社综合改革、农垦改革和高新区体制机制创新，统筹推进价格、财税、投资、公共资源交易等重点改革和经济发达镇行政管理体制改革，实行国有资产统一监管。抓好“两型”社会试验、服务业综合改革、小池全面深化改革示范区等改革试点。创新土地利用管理机制，创建国土资源集约节约示范市。

　　促进创新创业。扶持能人创业，激励全民创业。加强市校合作，提升“千企联百校”，推进科技孵化器市场化运营，新增孵化企业200家，新增一批众创空间，着力构建技术创新体系，打造“创天下”品牌。推进大别山英才计划，建立引得来、用得上、留得住的人才引进、培养和管理长效机制。聘用科技副总100名、引进创新团队50个，转化科技成果50项。引进2-3家股份制银行、3家以上金融分支机构。做好银企对接，创新金融服务，引导金融机构加大信贷投放。新设立一批银行业分支机构，推进全市金融服务网格化全覆盖。大力发展基金、债券等多层次资本市场，提高企业直接融资比例。引导省天使基金支持创业项目，实现创业投资引导基金投入运营。抢抓证监会开辟绿色通道支持贫困地区的窗口期，加大培育和引进力度，实现企业上市新突破。

　　激发市场主体活力。支持、引导非公有制经济发展，激发非公经济活力和创造力。落实国家支持市场主体发展的相关政策，大力实施市场主体成长工程、小微企业培育工程、新型业态成长工程、地理标志产品开发工程，实现新登记各类市场主体3万户以上，“个转企”500家。深入推进质量兴市、品牌强市战略，新增注册商标660件、著名商标22件以上。

　　扩大对外开放。积极对接大武汉，对接湖北自贸区，强化协作配套，承接疏解转移。推进大通关体系和一类水运口岸建设，谋划设立海关、商检等服务机构，对接“一带一路”，加强国际产能合作。积极融入长江经济带、长江中游城市群，持续推进跨江合作、环大别山战略合作，提高区域合作层次和水平。坚持全员招商与专业招商双轨并行，创新互联网+招商、精细化招商等方式，强化实绩考核，积极承接东南沿海地区产业转移，力争在产业链条关键环节和核心项目的引进上实现新突破。抢抓大型国有企业改革机遇，加强对接和合作，引进战略投资。

　　(五)以绿色发展为方向，厚植生态优势。绿水青山就是金山银山。绿色生态是黄冈的最大财富、最大优势、最大品牌，把好山好水保护好，把生态优势发挥好，是我们共同的责任。

　　坚持生态优先，构筑生态屏障。优化绿色发展空间布局，健全生态文明制度，加强山水林田路自然生态环境的保护与建设，创建大别山世界地质公园。启动大别山南麓天然林保护工程、林业碳汇工程，推进自然保护区和湿地公园建设，全面禁止天然林商业性采伐，持续开展“绿满黄冈”行动，推进退地还绿、拆围建绿、见缝插绿、人人护绿，共建绿色家园。继续推进“雷霆行动”，保护好母亲河。编制湖泊管理规划，全面推行“河长制”。实施全民节能行动计划，引导绿色消费，持续开展生态创建，让“红色历史、绿色风景”成为黄冈印象。

　　加大治理力度，提升环境质量。持续推进大气污染治理三年行动，减少大气污染源。狠抓秸秆露天禁烧，加强油品升级、城市扬尘、燃煤锅炉、工业废气、餐饮业油烟治理，完成黄标车提前淘汰工作。推进以治污水、防洪水、通下水、排涝水、保供水、抓节水“六水同治”及河湖连通为主要内容的“治水革命”。贯彻落实《黄冈市饮用水水源地保护条例》，禁止水库投肥投饵养鱼，开展畜禽养殖污染综合治理，加强重点水域生态修复，保障主要河流断面水质稳定达标和群众饮水安全。推进土壤治理改造，严厉打击非法开采矿产资源行为，抓好破损山体、采矿塌陷地治理，提高生态自我修复能力。实行“一控两减三基本”，开展化肥农药使用量零增长行动，让耕地休养生息。

　　加强资源利用，壮大绿色经济。强化约束性指标管理，实行能源和水资源消耗、建设用地等总量和强度双控行动。坚持保护、开发和利用有机结合，把绿色低碳改造作为重要抓手，全面推行清洁生产，大力发展循环经济，加快发展与生态环境相适宜的新产业新业态，努力实现环境指数与产业指标同步提升、经济与生态良性互动。支持团风绿色建筑产业发展，支持麻城建设绿色矿业发展示范区，支持英山、罗田打造绿色特色产业发展基地，支持龙感湖建设全国农村新能源集成基地。

　　(六)以脱贫攻坚为重点，增进民生福祉。无论财政压力有多大，民生支出只增不减，专项扶贫资金只增不减，为群众办实事的力度只增不减。

　　打好脱贫攻坚战。精准扶贫是黄冈最大的政治任务和第一民生工程，必须充分发挥政府、市场和社会协同作用，充分调动贫困群众的内生动力，实现稳定脱贫。突出产业扶贫，巩固扩大“千企帮千村”成果，推进“五位一体”产业扶贫落地生根。各贫困县市产业发展基金今年要达到1亿元以上，其他县市区达到5000万元以上。统筹抓好转移就业、易地搬迁、教育扶贫、健康扶贫、生态保护扶贫，特殊贫困群体要实现社会保障兜底。加大资金整合力度，着力改善贫困村生产生活条件。加大驻点帮扶、对口帮扶、社会帮扶工作力度，形成强大合力。加大督查考核和责任追究力度，实行第三方评估，杜绝“数字脱贫、被脱贫”，坚决完成318个贫困村出列、30万人脱贫的年度任务。

　　千方百计扩大就业。持续开展“春风行动”，加强技能培训，开发公益性岗位，加快发展建筑业、服务业等富民产业，城镇新增就业7.4万人，帮助2.3万名失业人员实现再就业。积极帮助高校毕业生、农村转移劳动力、城镇就业困难人员、零就业家庭等群体就业。以创业带动就业，强化创业培训、创业实训和创业孵化，实施“黄冈工匠”培育工程，加快高技能人才队伍建设。吸引10000名大学生到岗实习实训，引进创业团队200个以上。推进返乡创业计划，引导外出务工人员返乡创业。

　　健全社会保障体系。深入实施“全民参保计划”，推进机关事业单位养老保险制度改革，加快发展职业年金、企业年金和商业养老保险，完善失业、工伤、生育保险制度。全面实施城乡统一的城乡居民基本医疗保险制度，减轻群众看病负担。扩大农民工实名制管理范围，保障农民工权益。健全社会救助体系，发展社会福利事业。改善公共交通、通信、供水等基础设施，加快农村危房、城市棚户区和老旧小区改造，进一步提高安全饮水覆盖率、供水可靠率。关爱农村留守人员。健全困境儿童、高龄和失能老人、重度和贫困残疾人等特困群众保障及服务体系。加强社会保险、救助和慈善等方面资源统筹，兜住困难群体民生网底，让困难群众生存有尊严、生计有保障、生活有盼头。

　　加快社会事业发展。坚持教育优先发展，启动第三期学前教育行动计划，推进城乡义务教育一体化改革发展，深化高中教育改革，实施职业教育产教融合工程和质量提升计划。支持黄冈师院建设高水平综合性大学，支持黄冈职院创建国家级优质高职院校，继续实施黄冈中学振兴计划，加快职教园整合运行，进一步优化市区学校布局。大力推进“健康黄冈”建设，深化医药卫生体制改革，推动“三医联动”“三保”融合市级统筹，加强医疗卫生服务体系建设，改革基本医疗服务模式、健康服务运行机制、诊疗服务协作体系，提升人民健康水平。深入推进全民爱国卫生运动，积极创建健康城市、健康村镇。弘扬李时珍中医药文化，推动中医药振兴发展。实施“全面二孩”政策，完善计生服务管理。支持市疾控中心、妇幼保健院整体迁建。积极应对人口老龄化，加快城乡养老体系建设。加大国家“学生饮用奶计划”推广力度，扩大城乡和各类学校覆盖范围。推进全民健身，打造“15分钟体育健身圈”，筹办黄冈马拉松赛事。

　　(七)以社会和谐为目标，创新社会治理。回应群众关切，改进社会治理方式，为人民群众提供更便利、更均衡、更舒心的公共服务，让人民群众更有安全感、获得感和幸福感。

　　推进文化小康建设。以争创全国文明城市为载体，培育和践行社会主义核心价值观，开展群众性精神文明创建活动，提高公民素质和社会文明程度。加快构建现代公共文化服务体系，推进市、县文化基础设施建设，加强乡镇、村(社区)基层综合性文化服务中心建设。打造文艺精品，扩大黄冈文化影响力。加强红色文化、名人文化、戏曲文化、禅宗文化、孝善文化等历史文化遗产的保护利用，积极创建国家历史文化名城。大力发展文化产业，着力培育文化产业集群和特色文化产业强县。

　　健全公共安全体系。严格落实安全生产责任制，建立公共安全源头治理机制，健全事故灾难、暴力恐怖活动预警预防和应急处置体系，加强应急力量和应急装备建设，提升防范管控各类风险能力。开展全国食品安全示范城市和农产品质量安全县市创建，保障“舌尖上的安全”。

　　创新社会治理。加强系统治理、依法治理、综合治理、源头治理，健全有效预防和化解社会矛盾体制。完善阳光信访制度，加快信访法治化进程。创新维稳工作机制，健全重大事项社会稳定风险评估制度。持续开展信用年活动，推进社会信用体系建设，建立守信激励和失信惩戒机制。深入开展平安创建，加快立体化社会治安防控体系建设，依法防范和惩治各类违法犯罪活动，加强互联网安全管理，推进平安黄冈建设。全面推行网格化管理，提升社会治理水平。全面推进法治黄冈建设，深入实施“七五”普法，普遍建立法律顾问制度和公职律师、公司律师制度，完善法律援助、司法救助，让人民群众在法治中感受公平正义。

　　强化全民国防教育，加强国防动员、民兵预备役和人民防空建设，深入推进军民融合发展，支持驻黄部队建设，巩固军政军民团结。推进第三次全国农业普查工作，做好民族宗教、防震减灾、档案、气象和方志等工作。

　　四、努力建设人民满意政府

　　主动适应形势发展新要求，顺应人民群众新期待，秉持开明开放的理念、筚路蓝缕的精神、敢于争先的勇气、务实重行的作风，以实际行动诠释对党忠诚、展现为民情怀。

　　勇于担当，干事创业。发展是政府工作的第一职责。要以第一力度抓好第一要务，继续发扬拼的精神，始终保持干的状态，面对困难敢于迎难而上，面对危险敢于挺身而出，面对失误敢于承担责任，面对歪风邪气敢于坚决斗争，坚定不移抓好事关黄冈发展的大事难事。持续推进各级政府思想再解放、能力再提升、协作再增强，奋力实现开放再突破、改革再深化、发展再提速。鼓励创新，宽容失败，健全激励机制和容错机制，旗帜鲜明地为担当者担当、为负责者负责。

　　心系群众，执政为民。民之所望，政之所向。要矢志不渝坚持以人民为中心的发展思想，把执政为民的价值追求贯穿到政府工作的全过程，做到重大决策依民而定、工作措施应民而出、项目设施为民而建、安危冷暖唯民所系、发展成果由民共享。从解决群众所急所盼的问题入手，把精力放在群众衣食住行、业教保医、增加收入上，多办便民利民的实事、多做富民惠民的好事，以实干取信于民，以实绩造福于民。

　　依法行政，接受监督。将政府工作全面纳入法治轨道，严格在宪法和法律框架内行使职权，做到法定职责必须为、法无授权不可为。依法接受人大的法律监督和工作监督，自觉接受政协的民主监督，主动接受社会监督和舆论监督，认真执行人大及其常委会的各项决议决定，认真听取民主协商意见，切实办好人大代表建议和政协提案。坚持依法决策，严格遵守法定权限和程序，完善政府工作规则，进一步提高决策的科学化、民主化水平。坚持严格、规范、公正、文明执法。全面推进决策、执行、管理、服务、结果公开，运用全媒体手段及时回应社会关切，不断提高政府工作透明度和群众满意度。

　　改进作风，提升效能。坚持从一抓起、实干兴市，将目标落到项目、责任落到人头、措施落到实处，不急功近利，不邀功诿过，不做表面文章，不搞形象工程，力戒官僚主义、形式主义。坚持务实重行、踏实干事，把决策部署付诸于行动、体现于工作、彰显于业绩。坚持强化执行、跟踪问效，承诺的事情坚决兑现，既定的事情坚决办成。坚持转变职能、简政便民，少审批、少收费、优服务。开短会、发短文、讲短话。大力推进信息与政府管理服务融合，建设市县一体的“12345有事找政府”便民服务平台。加强乡镇服务能力建设，完善基层政务服务体系。

　　从严治政，保持清廉。坚定正确的政治方向，牢固树立“四个意识”。认真落实全面从严治党主体责任和“一岗双责”，深入推进政府系统党风廉政建设和反腐败斗争，保持良好的政治生态。深化“两学一做”学习教育，践行“三严三实”，贯彻执行两准则四条例，严格落实中央八项规定，坚持勤俭办事，保持“亲、清”政商关系，建设廉洁政府。完善财政资金支出、工程项目招投标、国有资产管理等制度，扎紧制度笼子，从源头上预防和遏制腐败。加强行政监察和审计监督，坚决纠正损害群众利益的不正之风，以零容忍态度惩治腐败行为。坚持严字当头，全面加强公务员队伍建设，锻造“两为”干部队伍，营造勤廉的政务环境。

　　各位代表，加快振兴崛起、决胜全面小康，承载着党中央、国务院的殷殷重托，承载着全市人民的共同梦想。实干托起梦想，奋斗铸就辉煌。让我们紧密团结在以习近平同志为核心的党中央周围，在省委、省政府和市委的坚强领导下，协力同心，锐意进取,为实现第一个百年奋斗目标谱写黄冈发展新篇章而努力奋斗!
